# Supplementary material for: Integration of residents’ experiences into economic planning process of coastal villages: Evidence from the Greater Hangzhou Bay Rim Area
Source: PLoS One. 2020 Oct 9;15(10):e0240125. doi: 10.1371/journal.pone.0240125 (PMC7546495; doi:10.1371/journal.pone.0240125)
Supplement: S1 File — (DOCX) [file pone.0240125.s001.docx]

**S1 File. Questionnaire in English**

**Survey on Rural Economic Development in Fishery Villages, Xiangshan**

| Time： | Site： | Investigator： | Questionnaire No： |
| --- | --- | --- | --- |

We are staffs in CML Engineering and Architecture School in Ningbo Institute of Technology, Zhejiang University. This survey is to examine the local economic development in fishery villages in Xiangshan, Ningbo. Your opinions will significantly contribute to the development of villages. This questionnaire will be conducted anonymously. It will take no more than 10 minutes to answer. Thank you！

**Section 1 Economic Environment**

（1-Strongly Unsatisfied 2- Unsatisfied 3-Average 4-Satisfied 5-Strongly Satisfied）

| No. | Questions | Strongly Unsatisfied Strongly Satisfied | | | | |
| --- | --- | --- | --- | --- | --- | --- |
| 1 | Fishery resources | 1 | 2 | 3 | 4 | 5 |
| 2 | Fish breeding industry | 1 | 2 | 3 | 4 | 5 |
| 3 | Impact of Tourism development on daily life | 1 | 2 | 3 | 4 | 5 |
| 4 | Local Job Opportunities | 1 | 2 | 3 | 4 | 5 |
| 5 | Current Working Status | 1 | 2 | 3 | 4 | 5 |
| 6 | Subsidy provided by government on Fishing and Farming | 1 | 2 | 3 | 4 | 5 |
| 7 | Dividends of village company | 1 | 2 | 3 | 4 | 5 |
| 8 | Household Income Satisfaction | 1 | 2 | 3 | 4 | 5 |
| 9 | Expectation of household income in 2018 |  |  |  |  |  |
| 10 | Growth of household income in last 5 years |  |  |  |  |  |
| 11 | Social Security |  |  |  |  |  |
| 12 | Numbers of family members |  |  |  |  |  |
| 13 | Number of family economic activities |  |  |  |  |  |

**Section 2 Social Information**

1. Gender： □ Male □ Female
2. Age：□ under 18 □ 18–25 □ 26–35 □ 36–45 □ 45–60 □ above 60
3. Education: □ Middle school or below □ High School Diploma (or high school equivalent)

□ Junior college or undergraduate degrees □ Postgraduate degrees or above

1. Profession: □ Self-employer □ Fisherman or farmer □ Retired □ Housewives

□ Student □ Technician or Professionals □ Migrant workers

□ Civil servant □ Enterprise Administrators □ Not stated

1. Household Income (Month Expenditure, CNY):
2. Numbers of family members: □ 1 □ 2 □ 3 □ 4 □ 5 □ more than 5
3. Number of family economic activities: □ 0 □ 1 □ 2 □ 3

□ 4 □ 5 □ more than 5
