# Supplementary material for: Integration of residents’ experiences into economic planning process of coastal villages: Evidence from the Greater Hangzhou Bay Rim Area
Source: PLoS One. 2020 Oct 9;15(10):e0240125. doi: 10.1371/journal.pone.0240125 (PMC7546495; doi:10.1371/journal.pone.0240125)
Supplement: S2 File — (DOCX) [file pone.0240125.s002.docx]

**S2 File. Questionnaire in original language**

**象山县渔农村村庄经济发展调查问卷**

| 调查时间： | 调查地点： | 调查员： | 问卷编号： |
| --- | --- | --- | --- |

您好！我们是浙江大学宁波理工学院土木建筑工程学院的老师，正在进行宁波市象山县渔农村经济发展的调查研究。非常感谢您能协助我们回答问卷的问题，您的回答对象山县渔农村美丽乡村建设非常重要。问卷采用不记名的方式，您的回答仅用于本次调查研究。回答问卷大约需要3分钟，我们对您的合作和参与再次表示由衷的感谢！

1. 经济环境

（1分为非常不满意，2分为不满意，3分为一般，4分为满意，5分为非常满意）

| 序号 问题 | 非常不满意 ———— 非常满意 | | | | |
| --- | --- | --- | --- | --- | --- |
| 1. 渔业资源 | 1 | 2 | 3 | 4 | 5 |
| 1. 养殖业发展 | 1 | 2 | 3 | 4 | 5 |
| 1. 旅游业发展对日常生活的影响 | 1 | 2 | 3 | 4 | 5 |
| 1. 当地工作岗位数量/工作机会 | 1 | 2 | 3 | 4 | 5 |
| 1. 目前的工作情况/环境 | 1 | 2 | 3 | 4 | 5 |
| 1. 渔农业经济补贴（渔业油价/农机等补贴） | 1 | 2 | 3 | 4 | 5 |
| 1. 乡村企业/集体收入 | 1 | 2 | 3 | 4 | 5 |
| 1. 2018年收入预期（养殖/种植/捕捞等收成） | 1 | 2 | 3 | 4 | 5 |
| 1. 家庭收入满意度 | 1 | 2 | 3 | 4 | 5 |
| 1. 过去5年的收入增长 | 1 | 2 | 3 | 4 | 5 |
| 1. 社会保障 | 1 | 2 | 3 | 4 | 5 |

1. 社会背景
2. 您的性别 □ 男 □ 女
3. 您的年龄 □ 18岁以下 □ 18 – 25岁 □ 26 – 35 岁 □ 36 – 45 岁

□ 45–60 岁 □ 60岁以上

1. 您的学历 □ 初中及以下 □ 高中/中专 □ 大专/大学本科 □ 硕士及以上
2. 您的职业 □ 学生 □ 个体户/私营业主 □ 企业管理人员 □ 专业技术人员

□ 离退休及待岗人员 □ 公务员/事业单位人员

□ 其他（请注明）

1. 您的家庭月均可自由支配（使用）收入： 元

（扣除税收、社会保险费用、预防意外开支的储蓄和生活必须消费等部分）

1. 您的家庭成员数量： □ 1人 □ 2人 □ 3人

□ 4人 □ 5人 □ 5人以上

1. 您的家庭主要劳动人数： □ 0人 □ 1人 □ 2人 □ 3人

□ 4人 □ 5人 □ 5人以上
