# Supplementary material for: Integration of residents’ experiences into economic planning process of coastal villages: Evidence from the Greater Hangzhou Bay Rim Area
Source: PLoS One. 2020 Oct 9;15(10):e0240125. doi: 10.1371/journal.pone.0240125 (PMC7546495; doi:10.1371/journal.pone.0240125)
Supplement: Attachment — Submitted filename: 20200713_rebuttal letter.docx [file pone.0240125.s003.docx]

Dear Editor, Dear reviewer

Thank you for your letter dated June 4. We were pleased to know that our work has merit for publication in Journal, subject to adequate revision. We thank the reviewer for the time and effort that he or she have put into reviewing the previous version of the manuscript. Your suggestions have enabled us to improve our work. Based on the instructions provided in your letter, we uploaded the file of the revised manuscript. Accordingly, we have uploaded a copy of the original manuscript with all the changes highlighted by using the track changes mode in MS Word. Appended to this letter is our point-by-point response to the comments raised by the reviewer. The comments are reproduced and our responses are given directly afterward in a different color (red). We would like also to thank you for allowing us to resubmit a revised copy of the manuscript.

We hope that the revised manuscript is accepted for publication in the Plos One.

Journal Requirements:

1. Please ensure that your manuscript meets PLOS ONE's style requirements, including those for file naming. The PLOS ONE style templates can be found at

https://journals.plos.org/plosone/s/file?id=wjVg/PLOSOne_formatting_sample_main_body.pdf and

https://journals.plos.org/plosone/s/file?id=ba62/PLOSOne_formatting_sample_title_authors_affiliations.pdf

Re: The manuscript has been revised according to PLOS ONE's style requirements.

2. Please include additional information regarding the survey or questionnaire used in the study and ensure that you have provided sufficient details that others could replicate the analyses. For instance, if you developed a questionnaire as part of this study and it is not under a copyright more restrictive than CC-BY, please include a copy, in both the original language and English, as Supporting Information.

Re: Both questionnaires (in English and in original language) have been provided as supporting information at the end of manuscript.

3. Please ensure that you include a title page within your main document. We do appreciate that you have a title page document uploaded as a separate file, however, as per our author guidelines (http://journals.plos.org/plosone/s/submission-guidelines#loc-title-page) we do require this to be part of the manuscript file itself and not uploaded separately.

Could you therefore please include the title page into the beginning of your manuscript file itself, listing all authors and affiliations.

Re: A title page has been added at the beginning of main document.

4. Please include your tables as part of your main manuscript and remove the individual files. Please note that supplementary tables (should remain/ be uploaded) as separate "supporting information" files.

Re: Tables have been added as part of main manuscript.

5. Please ensure that you refer to Figure 2 in your text as, if accepted, production will need this reference to link the reader to the figure.

Re: Figure 2(Fig 4 now) has been referred in the context.

6. Please upload a copy of Figure 7, to which you refer in your text on page xx. If the figure is no longer to be included as part of the submission please remove all reference to it within the text. We note there are two figures currently labelled as figure 1 in the figure files so please make sure that the figure labels are carefully checked and corrected as needed.

Re: All figures numbers have been rechecked to avoid misunderstanding.

7. We note that Figure 1 and 6 in your submission contain map images which may be copyrighted. All PLOS content is published under the Creative Commons Attribution License (CC BY 4.0), which means that the manuscript, images, and Supporting Information files will be freely available online, and any third party is permitted to access, download, copy, distribute, and use these materials in any way, even commercially, with proper attribution. For these reasons, we cannot publish previously copyrighted maps or satellite images created using proprietary data, such as Google software (Google Maps, Street View, and Earth). For more information, see our copyright guidelines: http://journals.plos.org/plosone/s/licenses-and-copyright.

We require you to either (1) present written permission from the copyright holder to publish these figures specifically under the CC BY 4.0 license, or (2) remove the figures from your submission:

7.1. You may seek permission from the original copyright holder of Figure 1 and 6 to publish the content specifically under the CC BY 4.0 license.

We recommend that you contact the original copyright holder with the Content Permission Form (http://journals.plos.org/plosone/s/file?id=7c09/content-permission-form.pdf) and the following text:

“I request permission for the open-access journal PLOS ONE to publish XXX under the Creative Commons Attribution License (CCAL) CC BY 4.0 (http://creativecommons.org/licenses/by/4.0/). Please be aware that this license allows unrestricted use and distribution, even commercially, by third parties. Please reply and provide explicit written permission to publish XXX under a CC BY license and complete the attached form.”

Please upload the completed Content Permission Form or other proof of granted permissions as an "Other" file with your submission.

In the figure caption of the copyrighted figure, please include the following text: “Reprinted from [ref] under a CC BY license, with permission from [name of publisher], original copyright [original copyright year].”

7.2. If you are unable to obtain permission from the original copyright holder to publish these figures under the CC BY 4.0 license or if the copyright holder’s requirements are incompatible with the CC BY 4.0 license, please either i) remove the figure or ii) supply a replacement figure that complies with the CC BY 4.0 license. Please check copyright information on all replacement figures and update the figure caption with source information. If applicable, please specify in the figure caption text when a figure is similar but not identical to the original image and is therefore for illustrative purposes only.

The following resources for replacing copyrighted map figures may be helpful:

USGS National Map Viewer (public domain): http://viewer.nationalmap.gov/viewer/

The Gateway to Astronaut Photography of Earth (public domain): http://eol.jsc.nasa.gov/sseop/clickmap/

Maps at the CIA (public domain): https://www.cia.gov/library/publications/the-world-factbook/index.html and https://www.cia.gov/library/publications/cia-maps-publications/index.html

NASA Earth Observatory (public domain): http://earthobservatory.nasa.gov/

Landsat: http://landsat.visibleearth.nasa.gov/

USGS EROS (Earth Resources Observatory and Science (EROS) Center) (public domain): http://eros.usgs.gov/#

Natural Earth (public domain): http://www.naturalearthdata.com/

Re: Figure 1(Fig 3 now) has been reproduced by using data from Natural Earth (public domain): http://www.naturalearthdata.com/. Figure 6 has been removed.

8. We note that Figure 5 includes an image of a participant in the study.

As per the PLOS ONE policy (http://journals.plos.org/plosone/s/submission-guidelines#loc-human-subjects-research) on papers that include identifying, or potentially identifying, information, the individual(s) or parent(s)/guardian(s) must be informed of the terms of the PLOS open-access (CC-BY) license and provide specific permission for publication of these details under the terms of this license. Please download the Consent Form for Publication in a PLOS Journal (http://journals.plos.org/plosone/s/file?id=8ce6/plos-consent-form-english.pdf). The signed consent form should not be submitted with the manuscript, but should be securely filed in the individual's case notes. Please amend the methods section and ethics statement of the manuscript to explicitly state that the patient/participant has provided consent for publication: “The individual in this manuscript has given written informed consent (as outlined in PLOS consent form) to publish these case details”.

If you are unable to obtain consent from the subject of the photograph, you will need to remove the figure and any other textual identifying information or case descriptions for this individual.

Re: The participant has been informed that she was in an image. But all authors agreed to amend the figure 5 (Fig 8 now) by a photograph without participants.

Reviewers' comments:
Reviewer's Responses to Questions
**Comments to the Author**

1. Is the manuscript technically sound, and do the data support the conclusions?
The manuscript must describe a technically sound piece of scientific research with data that supports the conclusions. Experiments must have been conducted rigorously, with appropriate controls, replication, and sample sizes. The conclusions must be drawn appropriately based on the data presented. 
Reviewer #1: No

Re: The manuscript has been modified with respect to literature review, methodology, analysis, and discussion according to the reviewer’s suggestion. Also, these revisions have been made to articulate the data and conclusions as followings.

2. Has the statistical analysis been performed appropriately and rigorously? 
Reviewer #1: No

Re: The methodology and rationality of data analysis has been modified to support the process of statistical analysis as followings.

3. Have the authors made all data underlying the findings in their manuscript fully available?
The [PLOS Data policy](http://www.plosone.org/static/policies.action#sharing) requires authors to make all data underlying the findings described in their manuscript fully available without restriction, with rare exception (please refer to the Data Availability Statement in the manuscript PDF file). The data should be provided as part of the manuscript or its supporting information, or deposited to a public repository. For example, in addition to summary statistics, the data points behind means, medians and variance measures should be available. If there are restrictions on publicly sharing data—e.g. participant privacy or use of data from a third party—those must be specified.
Reviewer #1: Yes

4. Is the manuscript presented in an intelligible fashion and written in standard English?
PLOS ONE does not copyedit accepted manuscripts, so the language in submitted articles must be clear, correct, and unambiguous. Any typographical or grammatical errors should be corrected at revision, so please note any specific errors here.
Reviewer #1: Yes

5. Review Comments to the Author
Please use the space provided to explain your answers to the questions above. You may also include additional comments for the author, including concerns about dual publication, research ethics, or publication ethics. (Please upload your review as an attachment if it exceeds 20,000 characters)
Reviewer #1: 1. The contribution to the body of scientific knowledge is poor. I am not convinced that it saying something particularly new about the rural planning and the local economic development.

Re: We are grateful for the suggestion. To provide a clear golden thread linking everything together, we have reorganized the narratives of this manuscripts by rewriting abstract, literature review, and discussion, as well as the subheadings of each part. More than 2000 revisions have been made.

This research attempts to explore the role of residents’ experiences in economic planning in rural areas, and to exam whether perspectives of residents could indicate both social and economic sustainability in rural development, especially in these economic dynamic areas. To be clearer on research background and in accordance with the reviewer concerns, we have added a brief description of the rural planning in China, as follows:

*According to the priorities of rural planning in Zhejiang, China (see Figure 1), residential perceptions are integrated into environmental and social dimension, such as the accessibility of public transportation, living environment, protection of heritages and historic buildings, features of cultural tourism projects, and others related to the feelings and perspectives of local residents. However, decision-makers pay attention to the industrial transformation, business management, and logistics management in economic facets. These economic goals and visions are determined with a top-down manner with limited discussion as to how residents’ experiences and satisfaction could continuously act as a representation of the local economic development.*

This indicate the gap between the national vision of rural economic development and the planning practices conducted by local planning authorities.

2. Unsophisticated methodology was considered and typical results were concluded.

Re: This research is conducted based on case studies though a combination of questionnaire, on-site observation and documentary analysis. First, to promote the validity of results and the rationality of case studies, the representative of both cases was reinforced by controlling variables, as following:

*According to the Yearbook of Xiangshan (2016), traditional agriculture and fisheries, the pillar of the local economy of Gaotang Island, has moved to the stage of modernization and touristization. (p11)*

*In Gaotang, there is no significant differences in population between 18 villages (see Figure 2). However, residents in Jingaotang gained a growing income because Jingaotang wharf is a marked route to tourist attractions on Hua`ao Island. Moreover, Jiangbei is the central and developed village where county government located, which could catalyse economic growth in Jiangbei to some extent. To mitigate the geographical and political implications in data analysis, both Jingaotang and Jiangbei were excluded in the list of case selection. (p11-12)*

*Figure 2 Economic development in Gaotang (Data from Yearbook of Xiangshan(2016), edited by first author)*

*Two villages were selected from 16 villages in Gaotang as two representatives of different stages of economic development, stage of traditional fisheries and beginning stage of tourism. According to the work of Gao(2015) in fisheries development and transformation in Zhejiang, local residents could suffer decrease on income through the industrial transformation from fisheries to tourism. Moreover, the delivery of facilities and properties for developing local tourism could not directly bring local residents’ income at the beginning stage.*

*Among the 16 listed villages, SML and LG peaked at rural property and annual income (per person) respectively (Figure 2). This indicates that SML has delivered an increasing number of properties for industrial transformation, and LG residents gained relatively higher salaries compared with other villages under current economic circumstances. SML and LG provide two different images of rural economic development. (P13)*

Also, the rationality of question design has been better explained by adding as followings:

*Questions were designed according to the synchronic (industrial chain, logistics management, job markets, economic policy, etc.) and diachronic aspects (industrial transformation, economic growth, changing capital goods, etc.) of economic planning in rural areas as previously discussed. (P13)*

Finally, the rationale of using regression analysis, especially a backward design, has been reinforced as followings:

*The data analysis was also conducted through a backward design of multiple regression analysis to explore the correlation between HIS and other variables. In this way, it is possible to understand the role of modest-sized set of potential variables based on their statistical contribution (Freund, Wilson & Sa, 2006) (P16).*

In general, as an empirical research, the methodology was enhanced in the aspect of case selection, research design, measurement, and analysis. The methodological approach was explained by answering what kind of data was collected and how these data were articulated. Also, the methods of data collection and analysis was elaborated through linking with existing studies in order to show why these methods suit our research objectives and questions.

3. Lack of justifications and information of the selected study area (two coastal villages).

Re: Case study is a main research approach conducted in this research. It is important to illustrate the benchmark of case selection which impact the reliability and validity of results. Thus, more information of selected study areas was provided, as followings (this has been stated above):

*According to the Yearbook of Xiangshan (2016), traditional agriculture and fisheries, the pillar of the local economy of Gaotang Island, has moved to the stage of modernization and touristization. (p11)*

*In Gaotang, there is no significant differences in population between 18 villages (see Figure 2). However, residents in Jingaotang gained a growing income because Jingaotang wharf is a marked route to tourist attractions on Hua`ao Island. Moreover, Jiangbei is the central and developed village where county government located, which could catalyse economic growth in Jiangbei to some extent. To mitigate the geographical and political implications in data analysis, both Jingaotang and Jiangbei were excluded in the list of case selection. (p11-12)*

*Two villages were selected from 16 villages in Gaotang as two representatives of different stages of economic development, stage of traditional fisheries and beginning stage of tourism. According to the work of Gao(2015) in fisheries development and transformation in Zhejiang, local residents could suffer decrease on income through the industrial transformation from fisheries to tourism. Moreover, the delivery of facilities and properties for developing local tourism could not directly bring local residents’ income at the beginning stage.*

*Among the 16 listed villages, SML and LG peaked at rural property and annual income (per person) respectively (Figure 2). This indicates that SML has delivered an increasing number of properties for industrial transformation, and LG residents gained relatively higher salaries compared with other villages under current economic circumstances. SML and LG provide two different images of rural economic development. (p13)*

4. Lack of in-depth analysis of the rural development, rural tourism, agriculture and industrial transformation.

Re: This research attempts to analyze the rural development through the lens of synchronic (industrial chain, logistics management, job markets, economic policy, etc.) and diachronic aspects (industrial transformation, economic growth, changing capital goods, etc.) of local economy, but also to highlight the social sustainability in industrial transformation. Moreover, we suggest that the rural economy should be examined by combining social and economic sustainability as an integrality rather than the absolute household income in these economic dynamic rural areas. Thus, we added further discussion on rural development as followings:

*Further, the agriculture and industry should be delivered gradually to keep its integrality and equality in rural development (Long, 2018). The result of data suggests that LG deliver fisheries with traditional manner which met difficulties in fishery resources and labours. This could lead to several in-depth questions on industrialization and transformation of local fisheries, including the delivery of breeding industry, the application of fishing technique and the training system on fishery technician. Low additional value and labour-intensive fishery products become a major source of income which reduces HIS in LG. By comparison, SML residents pay more attention to prospect of engaging tourism development although the tourism projects were delivered gradually and slowly. Their satisfaction on household income can be impacted by the working environment, workload, and regular income brought by the industrial transformation and “touristization” in coastal rural areas. Consequently, HIS could help to exam the feasibility and integrality of industrial transformation in rural areas, which could reinforce the social equality, social interaction and other issues relating to social sustainability (Wang & Ian, 2019). (p29)*

5. The magnitude of HI effect on resident’s experiences and changing economic environment which seems to be the main focus of the paper is not clear and unjustified.

Re: The magnitude of HI, based on official statistics, is one of key indicators in changing economic environment. However, we suggested that resident’s experiences on HI could also be significantly impacted by the working environment, the stability of income, public facilities developed in the industrial transformation, and other social factors. To promote the clarity of this part, we revised the discussion section as followings:

*Secondly,* *household income satisfaction (HIS) is* *a comprehensive measure of residents’ experiences in rural development in coastal areas. It can indicate the social trends of industrial transformation which could not be understand directly through statistical analysis of economic performance in coastal rural areas. In SML, the residents were more satisfied with their monetary income than their counterparts in LG although the average income of SML residents is lower than LG according to government report (Figure 2). The satisfaction may not, however, directly reflect current absolute income. According to the annual report from NOFB, in 2017, of total 16.88 million CNY fishery family net income in Ningbo, 13.08 million CNY (77.5%) was contributed by fishery industry (NOFB, 2018). This indicates that the tourism industry brought only limited financial benefits to the rural areas，which is also underpinned by the image of annual income (Figure 2). However, the delivery of local tourism impacted on the physical environment and public services, as well as enriching local residents in SML with a regular income. In 2012, more than 80 percent of SML residents became shareholders in a village owned company which conducted land lease transactions and promoted tourism development (Wang, 2013). SML residents could receive their dividends from land leasing (see* *Figure 6a). Moreover, a majority of residential buildings were re-built and* *refurbished along with the development of local tourism and homestay industries (see Figure 6b). It is also worth noting that facilities and public services, such as car parks, community parks, and bus stops, are more accessible in SML than in LG (Figure 7). The transport facilities gained, however, a certain amount of criticism from LG residents because there are no local no car parks and the bus stop is far away from residential area. This could explain why local residents and decision-makers showed willingness to delivering rural tourism to some extent. In fact, HIS could act as a measure of “thriving business”, which indicates social and economic sustainability as a unit in rural industrial development. (p27)*

*The fishery industry brings relatively higher income than tourism at this beginning stage, however, the workload and environment of homestay offices and fishing-boats is entirely different. Further, the agriculture and industry should be delivered gradually to keep its integrity and equality in rural development (Long, 2018). The result of data suggests that LG deliver fisheries with traditional manner which met difficulties in fishery resources and labours. This could lead to several in-depth questions on industrialization and transformation of local fisheries, including the delivery of breeding industry, the application of fishing technique and the training system on fishery technician. Low additional value and labour-intensive fishery products become a major source of income which reduces HIS in LG. By comparison, SML residents pay more attention to prospect of engaging tourism development although the tourism projects were delivered gradually and slowly. Their satisfaction on household income can be impacted by the working environment, workload, and regular income brought by the industrial transformation and “touristization” in coastal rural areas. (p29)*

6. The plausibility and relevancy of the results for changing economic environment of coastal villages.

Re: According to the national policy and vision of “thriving business” in rural areas, the economic development in rural areas gained prominence from politicians and academics, as followings:

*In 2018, The CPC Central Committee and the State Council stated Strategic Planning of Rural Revitalization (2018-2022) which highlighted the relevance of economic development in rural areas by putting “thriving business” in the first place of rural revitalization.* Local decision-makers have started to deliver rural projects, especially tourism projects and in so doing, have sought to garner a greater understanding of local dynamics by engaging with the perspectives of rural residents (Lin, Wang, and Li, 2019; Zhang, 2018). *(p2)*

Moreover, this study focuses on the most economic dynamic areas in China, eastern coastal areas, where government have introduced a package of policies to encourage investment. More information has been added to support the relevancy of the results as followings:

*The strategic planning of Hangzhou BRA, one of the most* *economically dynamic areas in China, was determined to develop regional economy by investing more than 1500 billion CNY. More than 120 projects, including ones within the energy, education, tourism, and cultural industry sectors, would be delivered to contribute to enhanced regional competitiveness and sustainability. The policy of Hangzhou BRA inevitably impacted on the industrial and infrastructure development in Gaotang Island to some degree. Thus, the economic circumstances remain dynamic in Gaotang Island, which brings plan-makers a question as to whether the local residents’ experiences could directly indicate the changing economic environment. Through so doing, practitioners would address the question as to whether rural development could contribute to the further development of the tourism and cultural industries. (p12)*

7. Economic status and growth in china (particularly in eastern and central regions) seems to be the main catalyst of both development and transportation infrastructure growth, how this variable might affect the result of this study.

Re: The economic development of both villages has political and geographical implications in eastern coastal areas of China. This has been indicated by suggesting as followings:

*The policy of Hangzhou BRA inevitably impacted on the industrial and infrastructure development in Gaotang Island to some degree. Thus, the economic circumstances remain dynamic in Gaotang Island, which brings plan-makers a question as to whether the local residents’ experiences could directly indicate the changing economic environment. (p12)*

As we suggested at the beginning of section 2

*“coastal rural areas have, compared with inland rural areas, witnessed unique economic development that has been considered to be more dynamic with respect to urban-rural migration patterns, but also industrial transformation and income increases (Wu, 2017; Liu, 2007; Lu, 1997)”*.

We attempted to discuss the role of residents’ perceptions within these economic dynamic areas, and offer solutions of economic planning where has dynamic economic catalyst.

8. The main scientific messages and findings are missing.

Re: The main scientific massages have been added or revised in the part of discussion and conclusion. Also, the scientific messages has been examined by relating to the existing literature, for instance:

*Plan-makers and decision-makers could evaluate the social equity of industrial transformation through examining HIS in rural development. The social aspect of rural development is relatively more important than economic growth compared with urban areas, which replicates the disparity of prosociality between rural and urban residents in developing counties. (p29)*

*The focus was on discussing the potential for residents’ experiences to act as a feasible and valid instrument by which to conduct rural economic analysis, especially to reinforce the social sustainability of rural development. (p30)*

*Moreover, whether the status of rural economy, or “thriving business”, can be evaluated comprehensively through the lens of residents’ experiences is a concept that has yet to be fully addressed. (p31)*

*We conclude that the small number of samples addressed within this paper is nevertheless important and a valid means by which to analyse the economic performance and history as a whole in coastal rural areas within Chinese context. HIS could act as an indicator of social and economic sustainability in rural development. (p32)*

Also, the manuscript has been revised by adding more necessary figures and references. An acknowledgement was added at the end of work. Thank you again for your precious time and suggestions.

Kind regards,

Authors
