# Supplementary material for: Integration of residents’ experiences into economic planning process of coastal villages: Evidence from the Greater Hangzhou Bay Rim Area
Source: PLoS One. 2020 Oct 9;15(10):e0240125. doi: 10.1371/journal.pone.0240125 (PMC7546495; doi:10.1371/journal.pone.0240125)
Supplement: Attachment — Submitted filename: Response to Reviewers.docx [file pone.0240125.s004.docx]

Dear Editor Bing Xue and Reviewers,

Thanks very much for taking your time to review the revised manuscript. We appreciate all your comments and suggestions. Please find our itemized responses in a different color (dark red) and our revisions/corrections in the re-submitted files.

Reviewers' comments:

Reviewer's Responses to Questions

**Comments to the Author**

1. If the authors have adequately addressed your comments raised in a previous round of review and you feel that this manuscript is now acceptable for publication, you may indicate that here to bypass the “Comments to the Author” section, enter your conflict of interest statement in the “Confidential to Editor” section, and submit your "Accept" recommendation.

Reviewer #1: All comments have been addressed

Reviewer #2: All comments have been addressed

2. Is the manuscript technically sound, and do the data support the conclusions?

The manuscript must describe a technically sound piece of scientific research with data that supports the conclusions. Experiments must have been conducted rigorously, with appropriate controls, replication, and sample sizes. The conclusions must be drawn appropriately based on the data presented.

Reviewer #1: Yes

Reviewer #2: Partly

This research highlights the relevance of residents’ experiences in economic planning which is less discussed compared with environmental and social facets. The perceptions of public and local community in the field of monetary income and economic development could help planning practitioners during the process of plan-making. However, the rationality of making decisions in rural development should still be reinforced in environmental, social, and economic dimensions as a collective unit (p6 line16). Based on the reviewer 2’s concern on the validity of conclusion, the conclusion has been modified to indicate the limitations of this study and the potential readers, as followings:

*We conclude that the small number of samples addressed within this paper is nevertheless important and a valid and alternative means by which to analyse the economic performance and history as a whole in coastal rural areas within Chinese context. HIS could act as an indicator of social and economic sustainability in rural development. The result of residents’ experiences replicates, but also helps to explain, the official data in annual industrial reports. This, ceteris paribus, can allow practitioners to conduct rural economic studies by investigating, comparing, analysing, integrating, and synthesising perceptions of local residents in coastal rural areas. Such an approach also give rise to research opportunities that focus on urban-rural interaction, social inclusion, and social inequality; all of which are already gaining growing attention from practitioners and scholars in the field of planning. Moreover, this research enables a process of plan-making on rural economic development by integrating perceptions from diachronic public participation and synchronic observation on physical environment. (p32 line 1)*

3. Has the statistical analysis been performed appropriately and rigorously?

Reviewer #1: Yes

Reviewer #2: (No Response)

4. Have the authors made all data underlying the findings in their manuscript fully available?

The [PLOS Data policy](http://www.plosone.org/static/policies.action#sharing) requires authors to make all data underlying the findings described in their manuscript fully available without restriction, with rare exception (please refer to the Data Availability Statement in the manuscript PDF file). The data should be provided as part of the manuscript or its supporting information, or deposited to a public repository. For example, in addition to summary statistics, the data points behind means, medians and variance measures should be available. If there are restrictions on publicly sharing data—e.g. participant privacy or use of data from a third party—those must be specified.

Reviewer #1: Yes

Reviewer #2: Yes

5. Is the manuscript presented in an intelligible fashion and written in standard English?

PLOS ONE does not copyedit accepted manuscripts, so the language in submitted articles must be clear, correct, and unambiguous. Any typographical or grammatical errors should be corrected at revision, so please note any specific errors here.

Reviewer #1: Yes

Reviewer #2: Yes

6. Review Comments to the Author

Please use the space provided to explain your answers to the questions above. You may also include additional comments for the author, including concerns about dual publication, research ethics, or publication ethics. (Please upload your review as an attachment if it exceeds 20,000 characters)

Reviewer #1: (No Response)

Reviewer #2: I just wondering why the discussion specifically focus on monetary income and local economy, while this paper has a potential conclusion related to the context of coastal villages, while economy side will be supported by other factors such as geographical uniqueness as well as environmental and social facets.
I am also concern about the conclusion which has been revised by the author. It is interesting if the role of the community in terms of economic planning process can be connected to criticize existing planning policy or even global issue of participatory/collaborative planning, innovative governance, and special interest tourism.

Although the economic side of coastal villages has geographical implications, the economic developments of villages within the coastal areas are still variable depend on their environmental, economic, and social facets. Based on existing studies, planners met difficulties in making economic planning of rural areas because of a lack of supporting evidences from the local community (p9 line 14). Also, “the engagement of local residents in rural economic planning remains variable, which has been to date discussed extensively in environmental and social facets of coastal village development.” (p7 line 3). In this research, we attempt to conduct case study of SML and LG which share similar geographical, environmental, and social circumstances, to see whether monetary income satisfaction has potential to manifest the local economic and industrial planning.

Despite the academic reason, there are also political facets of paying attention to rural economy. According to the policy of Rural Revitalization in China (p3 line 4), the focus of developing rural areas has shifted from ‘Beautiful Countryside’ to ‘Thriving Business’ because policy-makers find that the promotion on built environment could not contribute to the local job market and revitalize the shrinking rural areas (p3 line 13). In fact, this research is initiated based on our 8-years planning work and observation in rural China. We found that some coastal villages remain less developed although practitioners have developed the built environment of these villages in economically dynamic areas. This also contributes a global discussion that what is rural regeneration, beautiful countryside or thriving business, in developing countries.

Furthermore, reviewer raised an interesting point that the perceptions of local community have potential to criticize existing planning policy or even global issue of participatory/collaborative planning, innovative governance, and special interest tourism. This suggestion inspires us to explore the potentiality of linking residents’ experiences with regional and even global planning and good governance in future studies. We have revised the conclusion to reinforce the limitation of this study, but also make suggestions for further studies, as followings:

*Finally, it is noted that this study gained insight from the contest discourse of communicative turn in spatial planning to debate over the viewpoint that rural residents’ perceptions are diachronic, comprehensive, and informative. It particularly highlighted the role of residents’ experiences in the discussion of mid-term and long-term economic development in coastal rural areas which are deemed of importance under the national policy of Rural Revitalization. This study also provides a pilot case study to encourage theoretical and practical researches on identifying, understanding, and articulating the in-depth rationalities that lie behind residential experiences and substantial realities in economically changing and transforming coastal villages. The integration of public perspectives into planning process could also be used as empirical evidences for studies on Collaborative Planning. Further studies could be conducted to explore and elaborate the potential relationship between residential experiences and evaluation of regional economic planning and good governance. (p33 line 1)*

7. PLOS authors have the option to publish the peer review history of their article ([what does this mean?](https://journals.plos.org/plosone/s/editorial-and-peer-review-process#loc-peer-review-history)). If published, this will include your full peer review and any attached files.


If you choose “no”, your identity will remain anonymous but your review may still be made public.


**Do you want your identity to be public for this peer review?** For information about this choice, including consent withdrawal, please see our [Privacy Policy](https://www.plos.org/privacy-policy).

Reviewer #1: No

Reviewer #2: No

Also, all figures have been converted by Preflight Analysis and Conversion Engine (PACE) and re-uploaded.

Kind regards,

Authors
